# Shape Completion Using Deep Boltzmann Machine

**DOI:** 10.1155/2017/5705693

**Published:** 2017-07-19

**Authors:** Zheng Wang, Qingbiao Wu

**Affiliations:** School of Mathematical Sciences, Zhejiang University, Hangzhou, Zhejiang, China

## Abstract

Shape completion is an important task in the field of image processing. An alternative method is to capture the shape information and finish the completion by a generative model, such as Deep Boltzmann Machine. With its powerful ability to deal with the distribution of the shapes, it is quite easy to acquire the result by sampling from the model. In this paper, we make use of the hidden activation of the DBM and incorporate it with the convolutional shape features to fit a regression model. We compare the output of the regression model with the incomplete shape feature in order to set a proper and compact mask for sampling from the DBM. The experiment shows that our method can obtain realistic results without any prior information about the incomplete object shape.

## 1. Introduction

Shape completion is one of the most important tasks in the field of image processing. The goal of shape completion is to generate likely configurations of pixels for a missing region of the shape, given the rest of the shape. A lot of work has been done in the previous decades via a variety of methods on different conditions. For instance, completing the shape by constructing smooth curves [1], filling the missing area via the adjacent frames in a video [2], or performing the task through the repeated patterns in the image [3]. However, frequently there are no prior information about the position of missing area or we may lack the suitable context in the image for shape completion. This paper deals with this situation by the deep learning framework, a powerful branch of machine learning.

Approaches of machine learning have been successful in many other applications of image processing, such as segmentation, object detection, feature extraction, and inpainting. Among the various methods, generative graphical model, such as Markov Random Fields and Conditional Random Fields [4], has showed its ability to find the correlations of the neighboring pixels, which is important in the task of shape completion. Recently, deep architecture has demonstrated its potential to represent high level features in the image, often leading to better performance in many fields [5–7].

Deep Boltzmann Machine [8] is a multilayer generative model, which has the potential of learning internal representations that become increasingly complex. With the natural advantages of generative model, we can obtain the shape completion result by sampling from it [9, 10]. While sampling does quite a good job, we should acquire the prior information about the missing region, which is often impossible in many applications. To still obtain a realistic result, we should seek for the missing region automatically.

In this paper, we train a DBM and use its hidden activation combining with the convolutional shape features to fit a regression model. The regression model helps to set the mask for sampling, which corresponds to the missing region of the shape. While little work has been done before, our work shows that deep learning framework may afford an alternative competitive method for shape completion.

The remainder of the paper is structured as follows. In Section 2 we review the important deep architecture DBM, which is the foundation of our experiment. In Sections 3 and 4 we introduce the scheme for training the regression model incorporated with convolutional feature and setting the mask for sampling. In Section 5 we present methods for quantitative estimation. We provide the experimental evaluation in Section 6 and make a conclusion in Section 7.

## 2. Deep Boltzmann Machine

**Figure 1:**
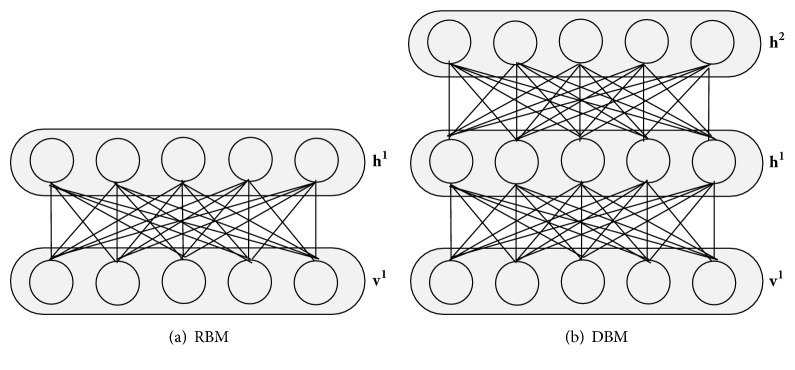
The structure of RBM and DBM.

A Deep Boltzmann Machine is a multilayer generative model which contains a set of visible units *v* ∈ {0,1}^*D*^, and a set of hidden units *h* ∈ {0,1}^*P*^. There are no intralayer connections. The DBM provides a richer model by introducing additional layers of hidden units compared with Restricted Boltzmann Machines, which are the building blocks of another deep architecture Deep Belief Network [11, 12]. We show the structure of these two models in Figure 1.

Consider a DBM with two hidden layers. The energy of the state {**v**, **h**^1^, **h**^2^} is defined as

(1) Ev,h1,h2;θ=∑ibivi+∑i,jwij1vihj1+∑jcj1hj1+∑j,kwjk2hj1hk2+∑kck2hk2,

where {*w*_*ij*_^1^, *w*_*jk*_^2^} are the connecting weights between layers and {*b*_*i*_, *c*_*j*_^1^, *c*_*k*_^2^} are the biases of each layer.

The probability of a visible state is

(2) $$\begin{array}{c} p\left(\;v;\theta \;\right)=\frac{1}{Z\left(\theta \right)}\underset{h^{1},h^{2}}{\sum }\exp\left(\;-E\left(\;v,h^{1},h^{2};\theta \;\right)\;\right), \end{array}$$

where *Z*(*θ*) = ∑_**v**,**h**^1^,**h**^2^_exp⁡(*E*(**v**, **h**^1^, **h**^2^; *θ*)) is the normalization constant. The conditional distributions over units in each layer are given by

(3) pvi=1 ∣ h1=σ∑jwij1hj1+bi,phj1=1 ∣ v,h2=σ∑iwij1vi+∑kwjk2hk2+cj1,phk2=1 ∣ h1=σ∑jwjk2hj1+ck2,

where *σ*(*x*) = 1/(1 + exp⁡(−*x*)) is the sigmoid function.

To learn the parameters of DBM, we try to maximize the log-likelihood log⁡*p*(**v**; *θ*) of the training data. It can be showed that the gradient of log-likelihood is

(4) ∂log⁡pv;θ∂θ=Eph1,h2 ∣ v∂Ev,h1,h2;θ∂θ−Epv,h1,h2∂Ev,h1,h2;θ∂θ.

The first term on the right side is the expectation of the gradient of the energy with respect to the posterior distribution over **h**^1^ and **h**^2^ given the training data **v**. This term is also called the data-dependent expectation. The second term on the right side is the expectation of the gradient of the energy respect to the joint distribution of **v**, **h**^1^, and **h**^2^. This term is sometimes called model's expectation.

In practice, exact computation of both the data-dependent expectation and the model's expectation is intractable. Instead, we prefer some approximation methods.

Salakhutdinov et al. deal with the difficulties via variational inference and MCMC [8, 13]. Variational approach is used to estimate the data-dependent expectation [14]. The posterior distribution *p*(**h**^1^, **h**^2^∣**v**; *θ*) is replaced by another distribution *q*(**h**^1^, **h**^2^∣**v**; *μ*) which can be fully factorized: *q*(**h**^1^, **h**^2^∣**v**; *μ*) = ∏_*i*=1_^*D*^*q*(*h*_*i*_), where *h*_*i*_ denotes the hidden units in **h**^1^ and **h**^2^, *D* is the number of all the hidden units, and *q*(*h*_*i*_ = 1) = *μ*_*i*_. Thus, the aim is to maximize the lower bound on the log-likelihood:

(5) log⁡pv;θ∑hqh ∣ v;μlog⁡pv,h;θ+Hq=log⁡pv;θ−KLqh ∣ v;μ||pv,h;θ,

where *H*(·) is the entropy and KL[·] is the Kullback-Leibler divergences.

The learning proceeds by maximizing the lower bound with respect to the variational parameters *μ*, or minimizing the KL divergences between the approximating and true posterior, equivalently.

To approximate the model's expectation, samples are drawn from the model distribution via Markov Chain Monte Carlo method [15]. Stochastic approximation procedure, also called Persistent CD, is used to generate samples [16–18]. In practice, we keep several Markov chains and use Gibbs sampler [15] while the states of the hidden units will be kept for next iteration. The conditional distributions required by Gibbs sampling are defined by (3). Many persistent chains can be run in parallel to better estimate the model's expectation [19, 20].

**Figure 2:**
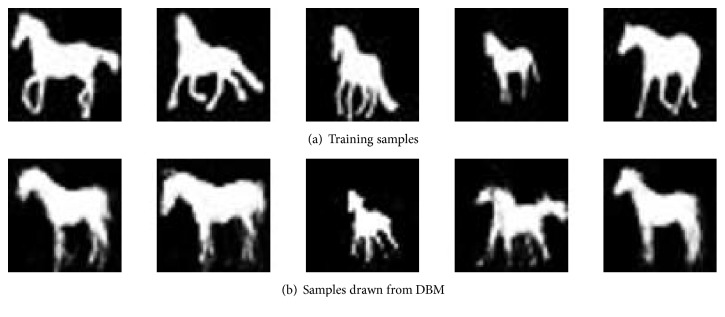
(a) Some training samples used to train DBM. (b) Samples drawn from the trained DBM.

It should be noted that the pretraining stage has important influence on the generalization ability of DBM [21]. Since there are often millions of parameters in the DBM, the gradient ascent in the likelihood may get stuck in local optima with a bad initial value, which will finally lead to a poor model. To avoid this, a greedily training method should be used to find an ideal initial value. We begin by training a Restricted Boltzmann Machine [22, 23] on the training data [24]. The training of RBM is very similar to DBM and is more simple compared with DBM, because the data-dependent expectation is tractable in this case. Then we use the activation of the hidden units in the first RBM as the input to train a second RBM. Finally, we use the parameters of the two trained RBMs as the initial parameters to train a corresponding two-layer DBM with the proper size. The whole training procedure is concluded in Algorithm  1. Some of the samples used or generated by DBM are shown in Figure 2.

## 3. Train the Regression Model

In this section, we describe the procedure of training the regression model, which plays an important role in seeking the appropriate mask for sampling. Recall that our goal is to complete the shape of one specific class, and we have no prior information about the mission part of the shape. Meanwhile, the DBM has powerful ability to capture the structure of the shape and is able to generate similar samples via sampling. On this occasion, we will naturally think of the method to compare the incomplete shape with the samples from the DBM.

An alternative method is to compare the above two shapes pixel-wise and set the mask with a threshold previously assigned. In other words, the mask indicates the region where the intensity of pixel changes drastically between the normal shape and the incomplete shape, which we assume is just the missing part we want. This plausible procedure rarely works well in practice due to the property of DBM. The DBM deals with the sample pixel by pixel and directly compares them which may result in an improper mask corresponding to a scattered missing area, which would lead to an unrealistic completion result. What is more, our task is shape completion rather than inpainting or denoising; it would be more appropriate to expect a smooth and connected mask for each missing part instead of the scattered one.

For shape completion, if the mask is restricted in a too small area, the sampling result would be unrealistic or even the shape would be separated. On the other hand, if the mask covers a broad area, the unimpaired shape in the original image would be largely affected and the task would fail. The ideal mask should have a proper scale; in other words, the mask should cover the missing region while having little effect on the rest normal shape.

Thus we propose an approach to overcome the difficulties. First we convert the original training sample to the convolutional feature set by some convolutional filter. To simplify the situation, we just make use of the mean filter of certain size. We then make another activation feature of DBM by a modified mechanism similar to the conditional sampling procedure in DBM. Recall that the state of the hidden layer is sampled by

(6) hj1~phj1=1 ∣ v,h2,hk2~phk2=1 ∣ h1.

The activation feature is acquired by

(7) hj1~=phj1=1 ∣ 2v,0,

(8) hk2~=phk2=1 ∣ h1~,

where $\tilde{h_{j}^{1}}$, $\tilde{h_{k}^{2}}$ are the obtained activation feature of each layer.

Attention need to be paid to these two formulas. In (7) we rescale **v** by 2**v** to approximate *p*(*h*_*j*_^1^ = 1∣**v**, **h**^2^) since the state of **h**^2^ is unknown. The sampling operator ~ is replaced by the assignment operator. For each sample in the training set, these two formulas are carried out only once.

Finally we fit a regression model with the activation feature and the convolutional feature. The object function to be optimized is

(9) $$\begin{array}{c} {\left\|\;XW_{r}+B_{r}-C\;\right\|}_{2}^{2}+\lambda {\left\|\;W_{r}\;\right\|}_{2}, \end{array}$$

where *X* is a matrix of *R*^*N*×*d*_*h*_^ and each row containing the activation of the hidden layer **h**^2^ is corresponding to each training sample. *W*_*r*_, *B*_*r*_ are the parameters of the regression model with sizes *R*^*d*_*h*_×*d*_*c*_^ and *R*^*N*×*d*_*c*_^. *B*_*r*_ is the duplicated version of the bias *b*, so that rows in *B*_*r*_ are identified with each other. *C* is the matrix of *R*^*N*×*d*_*c*_^, and each row contains the convolutional feature of sample. *N* is the number of training samples; *d*_*h*_, *d*_*c*_ are the dimensions of corresponding feature. *λ* represents the trade-off between the residual and the regularization item.

This optimization problem can be easily solved by stochastic gradient descent method. Thus we obtain the parameters *W*_*r*_ and *B*_*r*_, which are important in the following stage.

## 4. Completion with Mask

With the regression model described above, we are able to set an appropriate mask. Given an incomplete shape, we first run the traditional sampling steps for a few iterations, and, during the last iteration, we use the modified formulas to get the required activation feature *h*_*a*_ in hidden layer **h**^2^. We then reconstruct the convolutional feature by

(10) $$\begin{array}{c} c_{rec}=h_{a}\times W_{r}+b_{r}, \end{array}$$

where *b*_*r*_ could be any row in the matrix *B*_*r*_.

The mask is made by comparing the reconstructed convolutional feature with the convolutional feature *c*_ori_ obtained from the original incomplete shape, and we set the mask by

(11) $$\begin{array}{c} I_{M1}\left(\;x\;\right)=\left\{\begin{array}{cc} 1 & \left|c_{rec}\left(x\right)-c_{ori}\left(x\right)\right|>\tau \\ 0 & otherwise, \end{array}\right. \end{array}$$

where *I*_*M*1_(*x*) denotes the intensity in the mask, and *τ* is the threshold to control the difference.

Assume that the filter used in the convolution stage is of size *m* × *m*, and we denote *m*(*x*) as the *m* × *m* area which centers at pixel *x*. Thus we could enhance the mask by

(12) $$\begin{array}{c} I_{M2}\left(\;x\;\right)=\left\{\begin{array}{cc} 1 & x\in m\left(y\right),\exists y,I_{M1}\left(y\right)=1\\ 0 & otherwise. \end{array}\right. \end{array}$$

The whole procedure for setting mask is concluded in Algorithm  2.

Roughly speaking, the hidden activation contains the correlate information between the pixels in the visual sample, and *h*_*a*_ represents the abstract feature of the normal shape captured by DBM. In contrast, the convolutional feature represents the information directly from the visual space. Since our completion task is focused on the visual space, it is important to establish the relationship between these two different features, which is just the regression model's job. Comparing these two convolutional features is a reasonable way to compare the samples with the generative model.

The convolutional feature is essential in our approach and it brings mainly two kinds of benefits: for one thing, it affords some robustness to the procedure, and the comparison in the convolutional feature space is more reasonable than the pixel-wise comparison in the visual space. For another, the convolution operation maintains the region that we are interested in, and the procedure from *I*_*M*1_ to *I*_*M*2_ represents an inverse operation from feature space to visual space, which is of great concern in the task of shape completion.

To facilitate the completion, we clamp the pixels out of the mask and replace the pixels within the masked area with the sampling result from DBM.

While the mask may capture the proper area, it may also contain other small scattered regions which we should not pay attention to. We should keep the useful active area as well as discard the others. We thus resort to some morphology methods [25]. In order to achieve our goal, we use opening operation which erodes the image first and then dilates it. After this operation, the mask becomes more compact and consistent.

## 5. Quantitative Evaluation

While we could complete the shape via sampling, the probabilistic model defined by DBM may provide some quantitative information about the sample, which sometimes improves the result.

In the mean-field method discussed above, our aim is to maximize the lower bound on the log-likelihood:

(13) log⁡pv;θ∑hqh ∣ v;μlog⁡pv,h;θ+Hq=log⁡pv;θ−KLqh ∣ v;μ||pv,h;θ.

Using the factorized distribution defined in the previous section, the lower bound can be represented as

(14) log⁡pv;θ≥12∑i,jwij1viμj1+∑j,kwjk2μj1μk2−ln⁡Zθ+∑jμjln⁡μj+1−μjln⁡1−μj,

where *μ*^1^, *μ*^2^ are mean-field parameters for the corresponding hidden layer. Since ln⁡*Z*(*θ*) is constant when the parameter *θ* is fixed and it is unnecessary to calculate the lower bound precisely, we only pay attention to the remaining term.

For a new sample *v*^*∗*^ from the DBM, we run the mean-field fixed-point equations for a few iterations to update the mean-field parameters for the new sample:

(15) μj1=σ∑iwij1vi∗+∑kwjk2μk2+cj1,μk2=σ∑jμj1wjk2+ck2

and the convergence is usually fast. We then calculate the remaining terms in the lower bound, denoted by

(16) Rv∗,μ∗=12∑i,jwij1vi∗μj∗1+∑j,kwjk2μj∗1μk∗2+∑jμj∗ln⁡μj∗+1−μj∗ln⁡1−μj∗.

During sampling, we calculate *R*(*v*^*∗*^, *μ*^*∗*^) at every few steps of iterations, and replace the state *v*_1_ by *v*_2_ if *R*(*v*_1_, *μ*_1_) < *R*(*v*_2_, *μ*_2_). Otherwise we keep the state *v*_1_ as the initial state for the next few iterations.

It should be noted that *R*(*v*, *μ*) is the indicator of the lower bound rather than the real log-likelihood and we use *R*(*v*, *μ*) as a conservative strategy for the rationality of the completion result. If *R*(*v*_1_, *μ*_1_) > *R*(*v*_2_, *μ*_2_), we assume that log⁡*p*(*v*_1_) > log⁡*p*(*v*_2_). In other words, if *R*(*v*_1_, *μ*_1_) > *R*(*v*_2_, *μ*_2_), we think that *v*_1_ is more likely than *v*_2_ to be a suitable completion result, since *v*_1_ is more in line with the distribution of training samples.

The procedure of shape completion is described in Algorithm  3 as a summary.

## 6. Experiments

In this section, we demonstrate the completion results using the proposed method. We consider the Weizmann horse dataset as our training and test dataset. The Weizmann horse dataset (this dataset is publicly available and could be gotten from http://www.msri.org/m/people/members/eranb/weizmann_horse_db.tar.gz) contains 327 grayscale images of horses with various poses and sizes. The task is challenging because the model must capture the shape information with quite a small scale of dataset.

To train our DBM, we take 280 images as the training set while others test set. The images are normalized to 32 × 32 pixels as well as their convolutional shape feature. Thus the number of units in the input layer is 1024. We then train a DBM using the proposed method with 500,1000 units for layers **h**^1^, **h**^2^. The pretraining stage takes 400 epochs and the fine-tuning process takes 800 epochs. The total training procedure takes about 10 hours on an Intel Core i5 processor with 8 GB of memory running by MATLAB. We do not use GPU in the series of experiments.

We then use a mean filter of 33 pixels to make the convolutional features and train the regression model using stochastic gradient descent. We randomly pick the samples from test set and create some incomplete shapes manually. The incomplete shapes are used as the input for our completion method.

**Figure 3:**
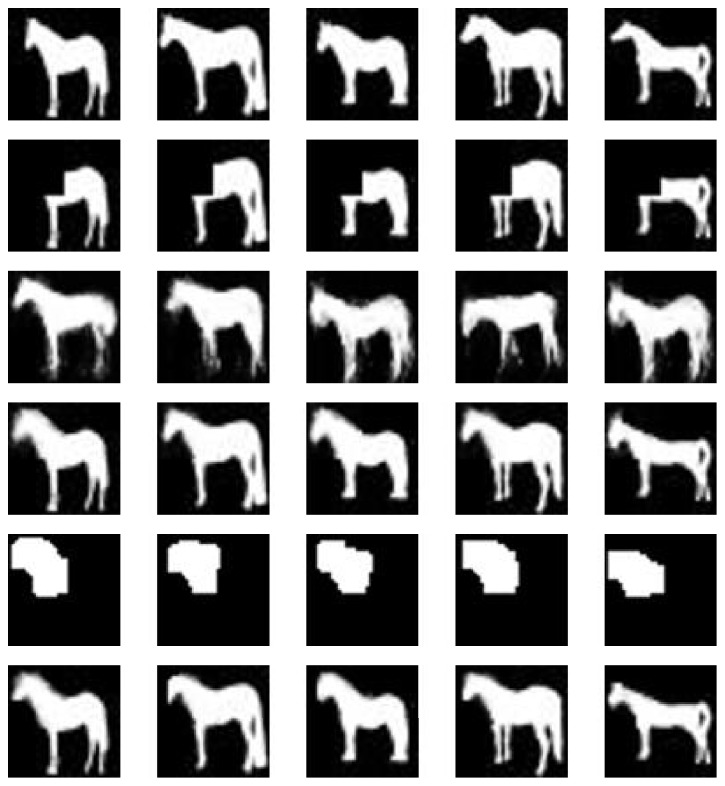
The completion result for some incomplete shapes. The original complete shapes are shown in the first row. The following rows represent the incomplete shapes, the completion result without any mask, the completion result with handmade mask, the mask found by the proposed method, and the completion result by our method, respectively.

We show our experimental results in Figure 3. Each column represents one same image in the different stages or result. The first row represents the test samples which have never seen before. The incomplete shapes are shown in the second row, which are made manually by setting the pixels of a quarter of the image to zero in the left above. The results in the third row are sampled from DBM where the initial visible states are just the incomplete shape, and no masks are used. We can find that although the shape in the image is complete, it changes a lot compared with the original shape and the completion is failed. For there are no constraints in the sampling procedure, any pixel in the image has some probability to change and the final result would get close to the samples drawn from the model distribution, while not keeping the original shape. The reason accounts for the usage of mask during the sampling procedure as well.

Thus we consider the next row in Figure 3. It represents the results where we use the manual mask. Since the incomplete shapes are made by ourselves, we have the prior information about the incomplete shapes. In other words, we can set the mask corresponding to the mission region exactly. The completion results reach the demand of reality and the shapes are similar to the original test samples. It is worth noting that this procedure is used to demonstrate the generative ability of the model in some related papers.

While the method of manual mask seems to meet the requirements to some extent, we should consider the case that appears more often where we have no prior knowledge about the mission region, which is the main objective in our work. Thus we must find the mask adaptively. The completion results by our method without any prior information are shown in the last row. Obviously, the completion results have not much difference between the two methods. Our method even generates more similar result to the test sample than the manual mask on some incomplete shapes such as the fifth column in Figure 3.

The result can be explained by finding mask with a proper scale. The masks found by our method are also shown in Figure 3. From that we see that each mask is constrained in the area near the head of horse, which is just the mission region we want to seek.

We could explain the completion result via the quantitative indicator *R*(*v*, *μ*). Values of *R*(*v*, *μ*) for samples in Figure 3 are shown in Table 1. The values of *R* for the incomplete shapes are much lower than the ones for the original shapes, since the incomplete shape is unfitted for the distribution modeled by DBM, which is a common result. We would pay more attention to the last three rows in the table. The value of *R* for the sample without mask is usually the highest one in the same column. Since there are no other constraints, the sampling procedure tends to obtain the result in the local optimum of the distribution modeled by DBM; thus the value of *R* is usually higher than other cases. As a result, samples without mask are easy to deviate from the original shapes or the incomplete shapes, which confirms the necessity of mask.

The values of *R* displayed in the fourth row indicate that the completion result is reliable from the point of probability, and, due to the mask, the completion result is also realistic from a visual standpoint. Finally, we focus on the result obtained by our method. It can be explained from two aspects. On the one hand, there is not much difference between the value of *R* for the original shape and the result through our method. It shows that our method can obtain quite reasonable result compared with the normal shape. On the other hand, the gap between the values of last two rows is limited, which demonstrates that our method has the ability to seek for a suitable mask without any prior information about the mission region.

**Figure 4:**
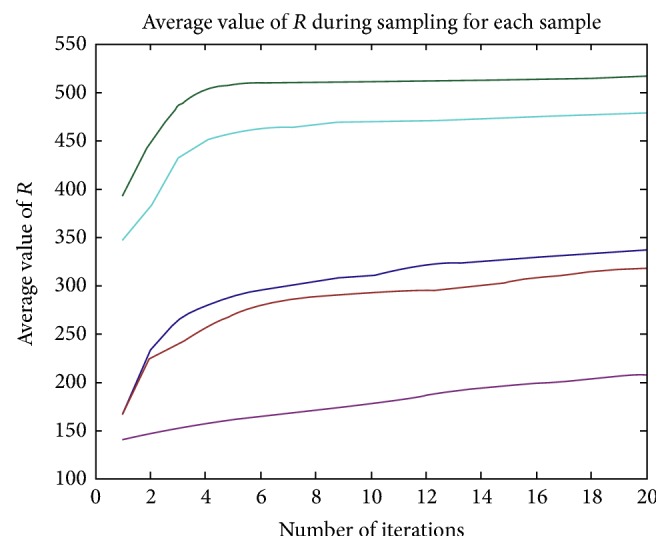
The average value of *R* for each sample during the shape completion procedure. For each incomplete shape in Figure 3, we run our method another 30 times and calculate the average value of *R* in each iteration. The five curves from top to bottom correspond to the average value of *R* for the sample in the second column, the fourth column, the first column, the third column, and the fifth column in Figure 3, respectively.

For each incomplete shape in Figure 3, we run our method another 30 times and calculate the average value of *R* in each iteration. The results are shown in Figure 4. We find that the values of *R* almost stayed constant during the last few iterations, and the final values are close to the values shown in Table 1, which indicates that our method is relatively stable. The time cost is negligible due to the few number of iterations. Some other completion results with different missing regions are shown in Figure 6.

There are some other considerations that need to be emphasized. We believe that a direct comparison with other method is not fair. On the one hand, different methods require different conditions. For instance, the method in [1] requires prior information about the exact position of the contour of the shape. Some other curve-based methods also require similar prior information. The method in [2] requires the adjacent frames in video. The method in [3] requires the similar patches of the object. Our method does not apply to those situations, and vice versa. In other words, different methods tend to apply only to certain special cases. We believe that it is unfair to compare different methods under different conditions. So we prefer to select the method under similar conditions to compare. On the other hand, there is a lack of a common and accurate performance measurement for shape completion. A large number of methods only visually evaluate the results, such as the methods in [9, 10]. The pixel-based performance measurement, such as the Euclidian distances between the corresponding points of the completed shapes and the ground-truth outlines (e.g., the mean value of the error for the horse class is 0.7021 with the Euler spiral method in [1]), may be inappropriate for our method. Because of the above reasons, we only compare the variations of the proposed method and use the value of *R* as a kind of quantitative evaluation.

To verify that our method could be used in other situations more than the Weizmann horse dataset, we train another DBM on the MNIST digit dataset (this dataset is publicly available and could be gotten from http://yann.lecun.com/exdb/mnist/). The MNIST digit dataset contains 60,000 training and 10,000 test samples of handwritten digits from 0 to 9 with the size of 28 × 28 pixels, which is a benchmark in the field of deep learning. It should be noted that we regard our training as an unsupervised procedure rather than supervised procedure, and we do not use the class label during training.

In this experiment, we use the raw pixels of each sample as the input data; the numbers of hidden units for the next two layers are 500 and 1000. Due to the abundant training samples, we reduce the number of iterations during training. The pretraining stage takes 100 epochs and the fine-tuning process takes 200 epochs. The procedure for regression model is similar to the procedure described above.

**Figure 5:**
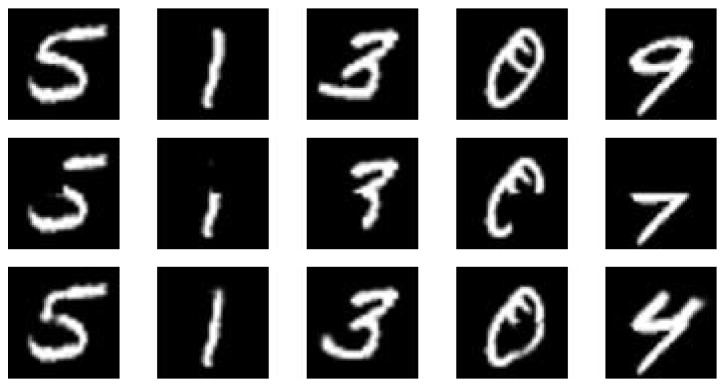
The completion result for digit.

**Figure 6:**
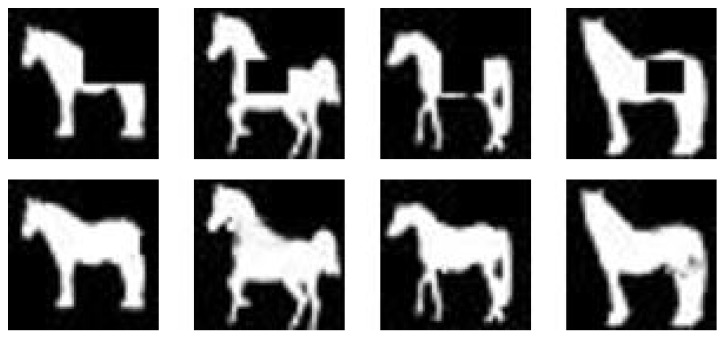
More other completion results.

The visual result and the term *R* are shown in Figure 5 and Table 2. We set the mission area corresponding to different four-quarters of the image in the first four samples and occlude the upper part of the last sample. We find that our method again obtains a comparatively satisfactory result. Something interesting has happened for the last sample. The completion result is the digit 4 rather than the original digit 9. We emphasize that we treat this completion result as a successful one. Recall that we assume we have no prior information about the missing region, and, for the case of multiple categories, this is equivalent to the case that we do not have the prior information for the class of the shape. In other words, the incomplete shape in the last column may come from a digit 9 as well as a digit 4, and the completion result is reasonable even the true digit is 9.

However, it should be emphasized that our method is highly dependent on the trained DBM with its model's distribution. The experiments also discovers that if the incomplete shape has a great difference compared with training samples, the task would be failed. This is because the shape deviates from the model's distribution greatly, and the procedure fails to estimate a proper mask for completion. From another perspective, our approach would be more powerful if the DBM is trained elaborately; for example, more training time or a more various and larger training dataset is provided.

## 7. Conclusion

In this paper, we have proposed a novel method for the task on shape completion via the powerful generative model DBM. In order to achieve better result, we train a regression model incorporating the hidden activation and the convolutional features, which plays an important role in the procedure of setting the proper mask. We search for an appropriate mask by sampling based on the mask from model's distribution. The experiments have shown that our method can achieve a satisfactory result without any prior information about the incomplete shape compared with manual method. Our work also demonstrates that the framework of deep learning may provide a competitive alternative method on shape completion.

## Figures and Tables

**Algorithm 1:**
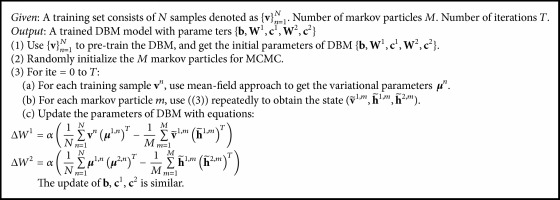
Training a DBM.

**Algorithm 2:**
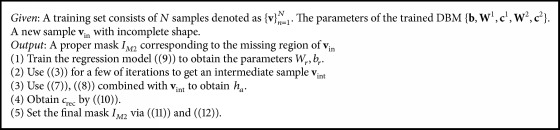
Find the proper mask for sampling.

**Algorithm 3:**
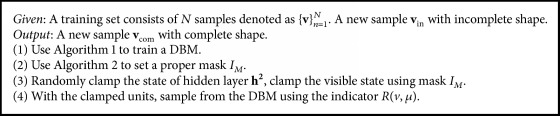
Shape completion.

**Table 1:** The value of *R*(*v*, *μ*) for each sample in Figure 3.

	Col1	Col2	Col3	Col4	Col5
Original sample	232.9193	548.6854	291.4416	471.6247	212.1185
Incomplete shape	167.0777	393.0472	169.7672	346.5666	140.9598
Sample without mask	500.2615	591.3720	461.5102	439.5120	458.3665
Sample with manual mask	370.2502	548.1915	391.8873	475.3915	297.2076
Result by our method	340.7963	521.8396	327.1240	471.8349	211.3393

**Table 2:** The value of *R*(*v*, *μ*) for each sample in Figure 5.

	Col1	Col2	Col3	Col4	Col5
Original sample	330.3212	207.6512	296.8532	228.7413	294.0450
Incomplete shape	281.9819	41.2760	120.6690	184.2102	127.0570
Result by our method	303.6035	191.1793	284.2993	234.2732	343.6164
